# Is quality of care during childbirth consistent from admission to discharge? A qualitative study of delivery care in Uttar Pradesh, India

**DOI:** 10.1371/journal.pone.0204607

**Published:** 2018-09-27

**Authors:** Malvika Saxena, Aradhana Srivastava, Pravesh Dwivedi, Sanghita Bhattacharyya

**Affiliations:** Research Department, Public Health Foundation of India, Gurugram, Haryana, India; University of Brighton, UNITED KINGDOM

## Abstract

**Background:**

Improving quality of maternal healthcare services is key to reducing maternal mortality across developing nations, including India. Expanding access to institutionalized care alone has failed to address critical quality barriers to safe, effective, patient-centred, timely and equitable care. Multi-dimensional quality improvement focusing on Person Centred Care(PCC) has an important role in expanding utilization of maternal health services and reducing maternal mortality.

**Methods:**

Nine public health facilities were selected in two rural districts of Uttar Pradesh(UP), India, to understand women’s experiences of childbirth and identify quality gaps in the process of maternity care. 23 direct, non-participant observations of uncomplicated vaginal deliveries were conducted using checklists with special reference to PCC, capturing quality of care provision at five stages—admission; pre-delivery; delivery; post-delivery and discharge. Data was thematically analysed using the framework approach. Case studies, good practices and gaps were noted at each stage of delivery care.

**Results:**

Admission to maternity wards was generally prompt. All deliveries were conducted by skilled providers and at least one staff was available at all times. Study findings were discussed under two broad themes of care ‘structure’ and ‘process’. While infrastructure, supplies and human resource were available across most facilities, gaps were observed in the process of care, particularly during delivery and post-delivery stages. Key areas of concern included **compromised patient safety** like poor hand hygiene, usage of unsterilized instruments; **inadequate clinical care** like lack of routine monitoring of labour progression, inadequate postpartum care; **partially compromised privacy** in the labour room and postnatal ward; and few incidents of **abuse** and demand for **informal payments**.

**Conclusions:**

The study findings reflect gaps in the quality of maternity care across public health facilities in the study area and support the argument for strengthening PCC as an important effort towards quality improvement across the continuum of delivery care.

## Introduction

In the past decade, maternal and newborn survival has been at the top of global health agenda, and plays a crucial role in the overarching health goals of the Millennium Development Goals (MDGs), and more recently, the Sustainable Development Goals 2030 (SDGs) [1]. As a result of concentrated global advocacy and action focusing largely on increased access to institutional care, the proportion of deliveries attended by skilled birth attendants in developing countries rose from 57% in 1990 to 70% in 2014 [2]. However, this increase has not translated into the desired reduction of maternal mortality, with an estimated 302, 000 deaths still occurring globally every year, almost 99 percent of which occur in lower and middle income countries [3]. Institutionalized care with increased access alone has failed to address the critical barriers in providing quality care that is safe, effective, patient-centred, timely and equitable [4,5].

Quality of care (QoC) encompasses the dimensions of structure, process and outcome of care, each aspect requiring equal emphasis for holistic improvement in QoC [6–8]. Person-centered care (PCC) is a critical component of QoC, as it focuses on the care that is respectful of and responsive to individual patient preferences, needs, and values and ensuring that patient values guide all clinical decisions [7]. PCC has been addressed using different terminologies such as patient, person, client, individual and are often used interchangeably in describing health care [9]. Recently there has been a shift from a care that supports disease-centred care to more on person-centred care where individuals are viewed as central to the decision making process and are free to exercise their rights as patients. Whereas the former type of care focuses primarily on the clinical aspect of the care giving less importance to other related aspects of care like respect; dignity, privacy, confidentiality, informed choices etc [10,11]. PCC is crucial for sustained utilization of maternal health services and to maximize health access and outcomes, especially in contexts of socio-economic, ethnic and cultural diversities [12]. The recent movement towards a composite approach in quality of maternity care has highlighted the need for improving the experience of women as an important dimension, despite this aspect of care being difficult to measure objectively [13]. The personal interaction between the woman and provider is important in shaping her experience and perception of clinical care [14, 15]. Women’s perspectives of maternity care that influenced their care-seeking behaviour ranged from poor staff attitudes; lack of amenities; perceived poor quality of health care facilities. Other reported barriers to effective utilization of maternity services were cost of care; poor access to health facility and health services being not available at odd hours [16–19]. Experience of verbal and physical abuse, negative provider attitude, poor information sharing and lack of emotional support from providers could be detrimental to utilization of maternity services [14, 20].

In particular, India has witnessed a sharp surge in institutional delivery from 39% in 2005–06 to 79% in 2015–16 with the implementation of the nationwide conditional cash transfer program—Janani Suraksha Yojana (JSY) under the National Health Mission (NHM) in 2005 [21]. Despite this, reductions in maternal mortality have been limited [21–24]. This is attributed to gaps in the clinical quality of facility-based maternal care [25, 26]. Recent evidence also points to women experiencing delays, neglect, abuse and poor client-provider communication during childbirth, leading to delayed care, and jeopardizing the likelihood of future utilization of facility-based care [21, 27–30, 31]. QoC in Indian policies and programs has fallen short at addressing the quality gaps in providing care that is more responsive to patient’s needs and ultimately leads to better outcomes [32, 33].

Few studies have examined continuity of care and assessed women’s experiences throughout her process of care. Consequently, we conducted direct, non-participant observations of the process of maternity care as experienced by women delivering in public health facilities in UP throughout their stay at the facility. The objective of the research was to understand the quality of care that was provided to women during childbirth in health facilities and identify associated gaps in the process of maternity care.

## Materials and methods

We conducted 23 qualitative direct, non-participant observations of uncomplicated vaginal deliveries in primary and secondary level public health facilities between October 2016 and February 2017. After completing 23 observations data saturation was achieved and it was decided to discontinue further data collection to avoid redundancy. The direct observation approach helped in gaining first-hand, in-depth information on the quality of delivery care, in addition to capturing complex and multiple patient-provider interactions.

### Analytical framework

**Fig 1:**
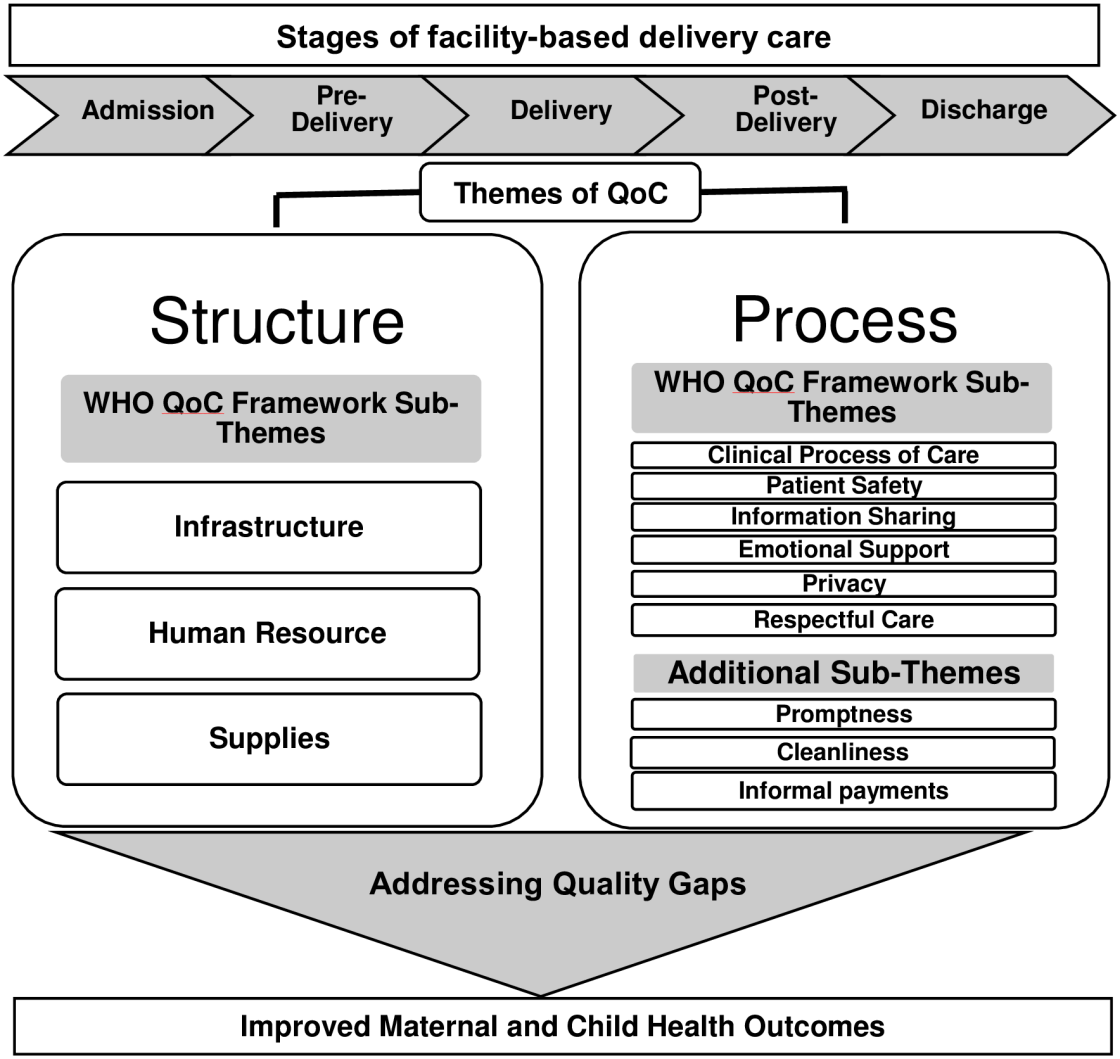
Analytical framework of QoC themes for facility-based maternity care across five stages of child birth. Broad themes of care ‘structure’ and ‘process’ in the analytical framework have been informed by Donabedian’s Quality framework. The WHO framework with its eight domains of quality of care informed the sub-themes. Three additional emerging sub-themes emerging from the data were included. The analytical framework was used for data analysis and guided the tool development. **Abbreviation:** QoC Quality of Care.

Our analysis is guided by Donabedian’s Quality framework and the World Health Organization’s (WHO) Quality of Care Framework for maternal and newborn health [6, 34]. The results section is organised based on Donabedian framework of broad themes of care ‘structure’ and ‘process’. Donabedian defines *Structure* as the organisational and professional resources associated with the provision of health care (e.g. staffing; facility infrastructure; availability of medicines and staff training) and *Process* as how the care is given ie the things done to and for the patient (e.g. technical quality; experience of care). The WHO framework with its eight domains of quality of care informed the sub-themes (Fig 1). *Infrastructure* refers to the physical structures like buildings including arrangements for water and electricity, *Human resources* refers to the number and configuration of hospital staff; their competency levels; level of supervision; staff training, *Supplies* refers to medical and non-medical equipment/supplies in the hospital, *Clinical process of care* refers to adherence to the standard operating procedures for providing health care to patients, *Patient safety* refers to provision of health care which is safe and minimises risks and harm to service users, *Information sharing* refers to the provider-client information exchange for the purpose of diagnosis and the determination of preference for treatment, *Emotional support* refers to the access to one’s own social and emotional support and the psychosocial support given by the provider, *Privacy* refers to observance of patient’s privacy from others at all time, *Respectful care* refers to the way provider treats his/her patients throughout the process of care. The additional sub-themes are defined as *Promptness* refers to how quickly the care is provided; reducing delays in providing care, *Cleanliness* refers to the general cleanliness maintained in the hospital, *Informal payment* refers to the incidences of providers asking for payment including cash; in-kind or any kind of gifts under the table. The analytical framework also guided the tool development.

### Study setting

The observations were conducted across nine public health facilities in two districts of UP, in northern India. UP is the most populous state of India, but has some of the poorest health indicators. UP has the second highest Maternal Mortality Ratio (MMR) and the third highest neo-natal mortality rate (NMR) in the country, 285 and 49 respectively [35], compared to 167 [36] and 28 at the national level [37]) respectively. The study districts of Kanpur Nagar and Unnao were purposively selected for ease of access as both are neighbouring the state capital, Lucknow. Kanpur Nagar has a high proportion of urban population (65.83%) while Unnao has much lower urbanization (17.1%) and is primarily a rural district [38,39]. Of the two, Kanpur Nagar fairs relatively better on the MCH indices with 76% institutional deliveries (69% in Unnao) and 79% deliveries assisted by skilled personnel (71% in Unnao) when compared to the state averages of 68% and 70% respectively [40–42].

### Study sites

A list of all public health facilities (CHCs and PHCs) with similar levels of care were obtained and a total of nine facilities were randomly selected. These facilities were previous sites of a large scale maternal health trial for the implementation of WHO Safe Childbirth Checklist. A list of range of primary and secondary level facilities was included with an attempt to understand providers’ and system-level challenges specific to both levels and ensure heterogeneity in the data for wider implications. [S1 Table]

Study facilities ranged from 4–30 bed units, 2–3 delivery tables; all labour rooms had electricity with backup; 5 out of 9 labour rooms had a functional new born stabilization unit. Filtered potable water and hospital meals were being provided in two and four health facilities respectively. With respect to the availability of human resources across facilities there were about a minimum of 1 to a maximum of 5 Lady Medical Officers; 4 to 9 staff nurse and ANMs and 1 to 3 other support staff like sweeper in position. The patient load for vaginal deliveries in the month of September 2016 ranged from as low as 110 to 257 with an average of 179 deliveries being conducted across the centres.

### Study instruments

A pre-tested facility observation checklist was used to capture women’s experience of labour and delivery care at five different stages of delivery care—Admission; Pre-Delivery/Initial Examination; Delivery; Post-Delivery and Discharge. The checklist, adapted from national public health standards and maternal and newborn health guidelines. The checklist was not pre-validated and had 114 items describing the normal sequence of actions that would follow at every stage of institutional delivery as per the Indian standards of care [43–45]. Checklist items reflected the content (technical competency of provider, availability of supplies etc.) as well as the process (information shared, emotional support, promptness) with a focus on elements of quality of person-centred care [S2 Table]. For instance to understand the process of care a checklist item inquired about whether initial examination was done within 15 minutes after registration; woman told how far along she was in labour; woman face any abuse during labour etc. The observer could enter yes/no for every observation point, in addition to documenting open-ended comments about significant observations regarding quality of care that would support in understanding the case in greater detail.

### Data collection

Data was collected by a four member team of two clinically trained nurses and two social scientists. The nurse focused on the clinical aspect whereas the social scientists were capturing person centred aspect of care. Prior to data collection, the investigators were trained on the technique of direct, non-participant observation, checklist contents and expected details to be captured. Training included practice observations using the checklist, on which the senior researchers gave feedback to improve quality and depth of data recorded. The investigators visited each facility for a period of 4–5 consecutive days to conduct the observations. The team was stationed at the facility from morning to evening; eight night-time deliveries were also observed. Data collection at each facility was initiated by a formal introduction with the Medical Officer In-charge (MOIC) to explain the purpose of the project and brief the MOIC about the intended data collection plan. This was followed by a facility tour for familiarization with the staff and facility layout.

Informed verbal consent was obtained from all relevant staff prior to data collection. In order to minimize inherent biases of observation on provider behaviour (Hawthrone effect), one to two observations were conducted without formal recording, however there is a possibility of researcher bias as observations of behaviour are subjective in nature. To reduce this effect a team of two trained nurses and two social scientists were asked to conduct observation for each case simultaneously and later the observation checklist was matched for any marked discrepancy. The providers were assured that data collection was anonymous and individual performance would not be reported or published. In order to minimize changes to specific aspects of behaviour, the providers were briefed broadly about the overall observation procedure without sharing details of individual checklist items.

Pregnant women and their attendants visiting the facility for childbirth were randomly approached and the purpose of the observation was explained to them in a manner that other people present in the room could not listen to the conversation. Informed verbal consent was then taken from participating women (and family members in some cases) prior to starting the observation. A door-to-door approach was followed, meaning the same woman was observed at all five stages of delivery care from admission through discharge. Each observation lasted for about 8 to 12 hours and the same woman was followed until the end of the day and the observation continued the next day to capture the discharge procedure. There were no refusals, though there were a few cases where women had to be dropped from the observation process, as they were referred out of the facility. In two emergency situations the investigators had to intervene in order to assist the staff with arranging for the delivery.

At the end of each day, the entire research team discussed and completed a final checklist after comparing data from individual checklists and coming to a consensus on final data points. Open-ended comments from all four members were carefully included in the final checklist. The completed and approved paper-based checklists were transferred to soft-form by the study team.

#### Ethical approval and consent to participate

This study obtained ethical approval from the University of California, San Francisco (153312) and the Institutional Ethics Committee of the Public Health Foundation of India (TRC-IEC-276/15). In which the study received ethics committee approval for obtaining verbal consent. Verbal consent was documented in a participant log maintained by the observation team. A written permission from the State Mission Director, National Health Mission was obtained for undertaking the study in the selected facilities. Approval was also taken from the Chief Medical Officers of the two districts before commencing data collection.

### Analysis

Based on our analytical framework, data from the checklist was organized under the two broad themes (structure and process) and then further categorized into sub-themes including: infrastructure; human resource; supplies; clinical process of care; patient safety; information sharing; emotional support; respectful care and privacy. Additional sub-themes (informal payment, promptness and cleanliness) emerging from the observations were also identified and included in the framework. Percentage observations for each checklist item were calculated using MS Excel 2010 which are discussed here as ‘few’ (below 25%); some (25–49%); many (50–74%) and most (above 74%). Content from open-ended comments and notes section of all the checklists was thoroughly reviewed and indexed using the study framework. Narratives from the observations reflecting critical quality gaps across different stages of delivery care were also identified and presented as case-studies.

## Results and discussions

The results are organized under two broad domains of quality, specifically ‘structure’ and ‘process’ for each of the five stages of care during childbirth. The first domain (‘structure’) encompasses the organizational aspects of care: infrastructure; supplies and human resource. The second domain (‘process’) describes the behavioural and technical aspects of care: promptness, emotional support, information sharing, clinical process of care, patient safety; cleanliness, informal payments and privacy. We describe the process of care across five stages from admission to discharge for all the 23 cases.

### Stage 1: Admission

A uniform admission procedure was followed across all nine facilities. The staff nurses on duty at the maternity unit were responsible for admission or referral of the women on the basis of preliminary physical examination. Women reaching the facility for delivery were usually accompanied by a Community Health Worker (CHW) and one or more relatives. The labouring women were taken straight to the labour room, where the nurse conducted a physical examination and determined whether the baby could be delivered at the facility or needed referral to a higher level facility. In the meantime, male attendant(s) (usually a husband), was directed to the registration counter located in the common admission area of the facility to obtain a registration slip. At night, the nurse issued the registration slip, as the registration counter was closed. In cases of referrals, a referral slip was provided; a mandatory written consent from the husband was obtained and an ambulance was arranged.

No wheel chair or stretcher was available for taking most women into the maternity unit and in few cases they had to be carried by their companions. The first physical examination included measuring cervical dilation, time of contraction and determining position of the foetus. While taking medical history, most providers also enquired about onset of labour and membrane rupture. As per the Government of India’s Maternal and Newborn Health national guidelines for all women in labour (cervix ≥ 4 cm) plotting contractions, Fetal Heart Rate (FHR), maternal pulse, color of amniotic fluid every 30 mins; plotting temperature, blood pressure and cervical dilation in cms every 4 hours is recommended. Although blood pressure, FHR was measured for some women however, pulse and body temperature were not measured even once for majority of the cases. The waiting time for admission for most women did not exceed 15 minutes. None of the women or companions were asked for informal payments at this stage.

### Stage 2: Pre-delivery

The second observation stage spanned from the time the women were admitted to the maternity unit until they were shifted to the labour room for delivery. The examination took place in the labour room as there was no separate examination room in maternity units of any facility. The maternity unit in all facilities had a common ward for prenatal, as well as postnatal women. Most women spent close to 6–7 hours or more in the maternity unit prior to delivery. During this period, women were mostly observed walking around, squatting or sitting on the floor in the lobby area outside the labour room. In the event that a woman needed to rest, she would use the beds in the postnatal (PNC) ward.

Monitoring the progress of labour was limited to conducting cervical dilation exam ranging from once (in the first one hour) to a maximum of four times prior to delivery. Most of the times the providers were called to check the progression when the woman faced increasing pain or contractions. Only a few providers washed their hands and cleaned the woman’s perineum before conducting vaginal examination. None of the providers were observed maintaining partograph or any record of labour progression and instead relied on cervical dilation exam also called the ‘two finger test’/’per vaginal’ test. Close to half the women observed were not monitored at all prior to delivery, after the first physical examination at the time of admission. After performing cervical dilation exam, providers briefly informed women and companions about how long it would take for the delivery to happen. Providers ensured partial privacy of women during physical examination as all examinations were performed in the labour room. Though screens were available in labour rooms of most facilities, they were not being used for maintaining inter-patient privacy. Most women were accompanied by at least one female companion during the initial examination, who stayed with them during the pre-delivery stage. However, in a few cases women were left alone and unattended for periods longer than 15 minutes prior to delivery. None of the women or companions were asked for informal payments prior to delivery. Box 1 reflects to the poor clinical process of care being provided to pregnant woman in one of the facilities under study.

**Box 1. Clinical process of care**

After conducting the previous delivery, the nurse was on the terrace (open area next to the maternity unit). Meanwhile, the woman (already present on the terrace) started vomiting profusely and within no time laid flat on the ground and delivered the baby. The nurse came running and said: “why didn’t you tell me; I am standing here only…you fool!” The baby kept lying on the floor for about 2 mins, after which the nurse and sweeper brought the supplies. CHW and Mother-in-law were standing beside the woman but none of them picked up the baby from the floor. The sweeper held the baby, cleaned it with a cloth, and placed the baby on the mother’s abdomen. After which she clamped and cut the cord using a blade, and the nurse gave an oxytocin injection to the woman.

### Stage 3: Delivery

The third stage of observation was that of the delivery. Only a few women had to wait for more than 15 minutes before being shifted to the labour room once they were ready for delivery (fully dilated). It was observed that majority of the labour rooms had two to three delivery tables and well-equipped with power back-up to enable night time deliveries during power cuts. Most deliveries were performed by the staff nurse, assisted by one or more staff and the CHW. In addition to the CHW, all providers allowed at least one female birth companion with the women during delivery who could be the mother/mother-in-law or sister/sister-in-law. Companions of nearly half of the women were asked to purchase medicines (mostly oxytocin) and other supplies from nearby pharmacies located outside the facility. These included, syringes, blade, sanitary pads, soap and cloth for wiping the woman and her baby. Delivery of each woman was conducted on a separate delivery table. Almost all providers supported normal delivery by providing perineum support and good coaching in terms of emotional, physical and informational support, resulting in few episiotomies, however only a few women were allowed to deliver in the position they desired. The providers in most cases did not help the women climb onto or get down from the delivery table and mostly supported by her companions. Some women were left unattended for more than 15 minutes in the labour room.

With regard to patient safety, providers in nearly all cases used sterile surgical gloves for examining the women, though only in a few cases they performed hand hygiene prior to that. In many cases blades (used for cutting the umbilical cord) were reused after washing in bleaching solution. Other instruments were washed under running water and seldom soaked in decontamination solution after use. Delivery tables were wiped after each delivery was performed with the cloth brought by the women without use of any disinfectant. Most providers disposed the contaminated waste in leak-proof containers. However, all sharps were either left on the table or thrown on the floor, to be cleared by the sweeper later on, while the women were still lying on the delivery table.

As per the Government of India’s Maternal and Newborn Health national guidelines 10 IU of oxytocin IM must be administered within 1 minute of birth for normal labour and delivery. However, in half of the cases women were administered a uterotonic (oxytocin 1ml to 3 ml) prior to delivery for inducing labour pains and all injections were given without disinfecting the skin. The umbilical cord was clamped using a sterile clip in case of most deliveries. Immediate uterine massage was given to most women following delivery of placenta. Assessment for complete removal of placenta and membranes, as well as for perineal and vaginal lacerations was not carried out for close to half of the cases. Likewise, immediate postpartum care including taking vital signs, administering antibiotics and palpating the uterus was not performed for more than half of the cases. Most women were not exposed unnecessarily at any point during delivery.

Most babies were wiped and covered using a piece of cloth brought by the women’s families; the nose and mouth were cleaned using the same cloth. The birth weight was recorded either immediately or at a later time for most babies delivered. Immediate skin-to-skin contact was established for close to two-thirds deliveries. Most providers did not check baby’s temperature within 15 minutes of birth.

A few women were physically and verbally abused (talking roughly, pushing, pulling, slapping on thigh) by the providers including assistant staff, in the process of delivery. For instance pulling/pushing the woman on the delivery table for adjusting position; shouting at her and/or slapping on the thigh when the woman doesn’t seem to follow the instructions given by the provider; giving undue fundal pressure; ask her to go to some other hospital if not willing to follow provider’s instructions. This would generally happen in spite of the presence of the companions and ASHA worker. A few instances of demand for informal payments were also observed, where the staff asked for money before handing the baby to the women and their companions. Box 2: reflections pointing to an instance of informal payment being demanded from the woman’s family before handing the baby to the mother.

**Box 2. Informal payment**

The nurse placed the baby on the weighing machine uncovered and un-wiped and demanded money from the mother-in-law (MIL). The MIL placed 25 INR on the machine but the nurse was not happy with it and said: “are you not ashamed of offering such a low amount of money to me?” Then the MIL placed 10 INR more and said: “we are poor people and we have only this much to offer.” The nurse got angry and left the baby and money on the machine and left the room saying: “I don’t need this money. If you are poor then keep this money as you will need it”.

Table 1 lists the important indicators performed by the providers during procedure and post-procedure for all the 23 cases that were observed across all nine facilities.

**Table 1:** Clinical and non-clinical indicators: Direct observation of women (N = 23) in study facilities.

Stages	Indicators
Pre- Delivery	1. Initial examination done within 15 minutes after registration **(Most)** 2. Provider evaluate blood pressure **(Some);** pulse **(Few);** temperature **(Few)** 3. Provider evaluate test for glucose in urine **(None)** 4. Provider evaluate test for haemoglobin **(None)** 5. Determine position of fetus (**Most)** 6. Determine fetal Heart rate **(Some)** 7. Care provider wash hands before examination **(Few)** 8. Cleaning of woman’s perineum before examination **(Few)** 9. Woman’s privacy maintained during the physical examination (Many)*$ 10. Woman informed about labour progression (Many)*$ 11. Have to wait at the observation ward (Many)*$
Delivery	12. Women left alone at any point (Some)*$ 13. Use box of sterile instruments for each delivery **(Some)** 14. Monitoring of labour-Record keeping in partograph (**None)** 15. Motivated woman to push the baby **(Most)** *$ 16. Administers uterotonic **(All);** before delivery **(Many);** after delivery (**Many)** 17. Performs uterine massage immediately following the delivery of the placenta **(Many)** 18. Someone present during delivery to provide support **(All)** *$ 19. Woman face any abuse during labour (**Few)** *$ 20. Takes mother’s vital signs 15 minutes after birth **(Some)** 21. Palpates uterus 15 minutes after delivery of placenta **(Some)** 22. Disposal of all contaminated waste in leak-proof containers **(Most)** 23. Disinfect cord **(Most)** 24. Immediate skin-to-skin contact **(Many)**

*^$^ -Non Clinical indicators.

The table lists critical clinical and non-clinical indicators performed by providers for all 23 cases that were observed. These have been classified as ‘None (0%); few (below 25%); some (25–49%); many (50–74%); most (above 74%) and all (100%) and discussed under two stages of—pre-delivery and delivery.

### Stage 4: Post- delivery

The fourth stage of observation spanned from the time the women were shifted to the PNC ward until their discharge. In all cases women were kept under observation in the labour room for 45 to 60 minutes after delivery. They were examined for bleeding and shifted to the PNC ward, once their condition was stable. During this time the companions would arrange for tea and snacks (mainly biscuits) for the women and staff. The PNC stay for most women ranged from 6–10 hours in case of day time deliveries and up to 24 hours in case of late night deliveries. The PNC ward of most facilities was located close to the nurse duty room and facilitated monitoring of women in the ward.

PNC wards in all the facilities were equipped with beds, electricity (with backup), toilets and drinking water supply mostly hand-pumps. There was no restriction on the number of visitors or companions allowed inside the PNC ward, often making the ward too crowded during visiting hours. The presence of male visitors in the PNC ward impeded privacy during breastfeeding. Although all women and babies got a separate bed during their stay in the PNC ward, there was no arrangement for companions, who either shared the bed with the women or slept on the floor. The bed cots and bedsheets were clean in almost half of the facilities. However, toilets were found to be dirty and the light fixtures were often missing or faulty. Hospital meals for the women were available in close to half of the facilities.

Staff worked in shifts of 10–24 hours, ensuring that at least one staff member (Nurse/Skilled Birth Attendant/Doctor) was available in the maternity unit most of the time. Close to half of the women were visited by a nurse or doctor at least once during their stay. The examination was limited to verbal enquiry of general condition of the baby and mother, and checks of blood pressure and postpartum bleeding. Only some women received counselling on essential postnatal care that focused mainly on exclusive breastfeeding, immunization and family planning. The number of women counselled for danger signs of mother’s and baby’s health were very few. A few women faced verbal abuse (talking roughly) during the postnatal period. Few providers were seen demanding informal payments. Box 3: reflections pointing to an instance of poor post-delivery newborn care provided to one of the cases being observed.

**Box 3. Post- delivery care**

It was a breech baby. The baby was born with some deformity in the feet and did not cry soon after birth therefore the nurse started the resuscitation process. The baby was shifted to the Newborn Stabilization Unit and was placed in a baby warmer. The nurse started reviving the baby with the help of ambubag. She tried to inject 5 ml calcium gluconate mixed in 5 percent dextrose in the cord of the baby but the cord clip was clamped too close to the navel of the baby leaving no space for injecting the drug. When injecting the drug through the cord failed, the doctor (on emergency duty) advised for injecting the drug via catheter. It took almost close to 10 minutes for the nurse to find a surgical tape before preparing a catheter opening, which also did not work. Next, the team started aspiration using mucus extractor. The baby could not be revived and seeing the baby unresponsive the doctor decided to refer the baby to the nearby district hospital but soon the baby died. After completing the final investigation the doctor informed the family and mother about death of the baby.

### Stage 5: Discharge

The fifth stage of observation was the discharge process, in which the women filled the Janani Suraksya Yojna form to claim their financial incentive. At the time of discharge the staff usually vaccinated the babies, counselled the women on postnatal care and dispensed medicines. Across facilities, the staff nurse completed most of the discharge formalities except JSY paper work and arrangement for transport, which was done by the CHWs. A few women were counselled at their bed side. All others were counselled in the nurse duty room while the baby was being vaccinated. The counselling mainly focused around immunization; family planning and exclusive breastfeeding. Identifying the baby’s and mother’s danger signs was the least discussed topic. Most women and their companions were given information on the immunization schedule, whereas only some of them received instructions for follow-up check-ups. Close to half of the women did not receive free medicines and cotton/sanitary pads at the time of discharge. Informal payments were demanded from a few women at the time of discharge.

## Discussion

This paper describes the findings of the quality of maternity care provided in primary and secondary level public health facilities in two districts of UP, India. Direct, non-participant observations were used to capture the critical components of both clinical as well as non-clinical obstetric care from admission through discharge. Door-to-door observation of the entire journey of a woman’s experience of delivery care allowed identification of critical gap areas specific to each of the five stages. Few other studies have similar in-depth documentation [22, 27, 46–48]. These studies were conducted in similar settings—Ethiopia; Nigeria; UP and MP in India and most of them used direct observations of intrapartum care provided during labour and childbirth except one in Nigeria that employs a different qualitative approach to capture the experiences of providers and women. Good practices, as well as gaps, were observed at each stage of delivery care. Most gaps were noted during delivery and pre-delivery stage, most strikingly on patient safety and the clinical process of care. Overall, though human resources and infrastructure were available across all facilities, five key areas of concern were identified: inadequate clinical care and patient safety, information sharing, compromised privacy, disrespectful care and informal payments.

Table 2 summarizes key good practices and areas of concern noted in the observations across five stages of delivery care. Most gaps were identified during the ‘delivery stage’. ‘Clinical process of care’ and ‘patient safety’ during pre-delivery and delivery stages were the key areas of concern where gaps were noticed in the quality of care provision. Gaps were observed in other areas of care provision that included information sharing, maintaining privacy, providing emotional support and demanding informal payment. Structural gaps—infrastructure and human resource provision were not as startling as the areas included in the ‘process’ of care.

**Table 2:** Summary of areas of concern and good practices observed in the study.

Themes of care	Good practice	Areas of concern
**Infrastructure**	**Admission** Signboards showing registration counter; Ramp way clear; defined waiting area with seating arrangement; drinking water and power supply (with backup) **Pre-delivery** Adequate beds and delivery tables **Post-delivery** Adequate beds available for all women and babies **Discharge** Free transport for home arranged	**Admission** No stretcher or wheelchair provided to take women to labour room. **Post-delivery** Adequate seating and sleeping arrangements not available for companions
**Human Resource**	**Delivery** All deliveries conducted by skilled provider (mostly staff nurse)	**Post-delivery** Security guard not present to regulate visitors in PNC ward
**Supplies**	**-**	**Delivery** Family members arranged for medicine/cotton **Discharge** Women did not receive medicines; pads/cotton
**Clinical Process of Care**	**Delivery** Sterile surgical gloves used for examination of most women	**Pre-delivery** Incomplete initial examination—blood pressure; pulse; temperature; hemoglobin and glucose in urine and fetal heart rate not conducted for close to half of the women Delivery Partograph not filled during and after delivery Administration of uterotonic (oxytocin 1ml to 3 ml) prior to delivery—inducing labour pains Skin not disinfected before giving injection; Perineal and vaginal lacerations not assessed; Vital signs not taken 15 minutes after delivery; Uterus not palpated 15 minutes after delivery of placenta; Immediate skin-to-skin contact not established Vaginal packing and giving unwarranted fundal pressure (Most cases) For most babies, temperature and skin color not monitored 15 minutes after birth and vitamin K not administered Lining the uterus with mustard oil before delivery (Few cases) Cleaning the baby’s body and inside of the mouth with mustard oil (Few cases) Keeping the baby uncovered until cord cutting and weighing (Few cases) While using the mucus extractor Mothers asked to suck from one end while inserting the tube in baby’s mouth and nostrils (Few cases) **Post-delivery** No examination conducted (including blood pressure) and most babies were not examined in PNC ward
**Patient Safety**	**Delivery** Disposal of all contaminated waste in leak-proof containers	Pre-delivery Hand hygiene and cleaning women’s perineum before examination nearly absent Using condom for conducting per vaginal examination (Few cases) Used gloves left on the delivery table close to the patient (Few cases) Delivery Hand hygiene not performed before any examination; sharps not disposed properly and immediately after use; sterile supplies (gloves, cotton; blade; tray) not used for some deliveries The broken (used) oxytocin vials were thrown on the floor
**Information Sharing**	**Pre-Delivery** Women were told how far they were in labour	**Pre-delivery** Women or family members not able to ask question to the providers Post-delivery Information about mother’s and baby’s health not shared with companions; Counseling on breastfeeding and thermal care not provided Danger signs not discussed **Discharge** Women were not counselled on family planning; immunization; exclusive breast feeding; baby’s and mother’s danger sign and instructions for follow-up check-ups not given
**Emotional support**	**Delivery** Female companions allowed in labour room **Post-delivery** Companions stayed throughout in the PNC ward	Delivery No support in helping women climb on the delivery table; Women left alone in the delivery room post-delivery for more than 15 mins
**Privacy**	**Delivery** Most women not exposed unnecessarily during delivery	**Delivery** Dividing screen/curtain between delivery tables not available in labour rooms **Post-delivery** Male visitors were present in the PNC ward at all times
**Respectful Care**	**-**	**Delivery** Women faced verbal and/or physical abuse during labour (few cases)
**Promptness**	**Pre-delivery** PV and abdominal examination conducted within 15 minutes of reaching the facility for all women Post-delivery Women were kept under observation in labour room for more than an hour before shifting to PNC ward JSY paper work completed before discharge	**Post-delivery** Women not visited by provider at least once in three hours
**Cleanliness**	**-**	Post-delivery Cots/bed sheets were not clean Toilets and bathrooms not clean
**Informal Payments**	**Pre-delivery** No provider asked for informal payment	**Delivery** Providers asked for money to the accompanied person (few cases) **Post-delivery** Out-of-pocket expenditure for services including medicines; informal payment; photograph for JSY (few cases)

**Abbreviations:** PV Per Vaginal; PNC Post Natal Car; JSY Janani Suraksha Yojana

The table lists ‘good practices’ and key ‘areas of concern’ as per the Indian Public Health Standards guidelines for Primary and Community health centres, 2012. These have been discussed under five stages of delivery care—admission; pre-procedure; procedure; post-procedure and discharge and further categorized into themes of quality care framework.

First, there were a number of gaps in clinical process of care. Study findings suggest that routine monitoring of labour such as use of partograph, fetal heart rate monitoring, blood pressure, temperature and pulse rate were not being performed by all providers uniformly. Providers in some cases administered uterotonic prior to delivery for inducing labour pains [49, 50], which is not consistent with national and global guidelines [51]. Similarly, some of the essential care practices during third stage of labour and immediate post-partum period like controlled cord traction, palpation of uterus, and assessment for perineal and vaginal lacerations, were not performed for most cases. This has also been observed in other studies [52–54]. According to a hospital-based cross-sectional study conducted in Netherland the active third stage management was being adequately performed in only close to half of all vaginal deliveries [54]. Other gaps include inappropriate maternal and newborn infection management, not providing immediate skin to skin contact and no assessment of newborn’s health [22, 46, 55]. Gaps in patient safety included providers not performing hand hygiene before wearing sterile gloves, instruments not being sterilized; and improper disposal of sharps. Similar evidence has been found in other studies conducted in India [22, 55, 56].

Lack of information, privacy, and emotional support were the other key gap areas in the process of care. Patient-provider interaction and information sharing was limited in terms of content, quantity and quality. Establishing a two-way communication with the woman, making her a receptive and active participant in improving the process of care, is often a neglected aspect of delivery care provision [28, 57, 58]. Other studies in conducted in Malawi and Haiti have observed sharing inadequate information on postpartum care, specially information on danger signs [59, 60]. In terms of maintaining privacy, though entry of males was restricted in all labour rooms, use of drapes and screens for providing privacy to the labouring women was not practiced, corroborating findings from other studies [27, 29]. Findings from a five country study conducted in health facilities in Africa points out women experiencing compromised auditory and visual privacy resonating well with our findings [27].

Recent reports from many low- and middle–income countries suggest that women are being denied labour companionship, especially during childbirth [27, 28]. However, we did not find any such restrictions in our study and our findings were consistent with other studies in India [18, 29]. All women were allowed to have at least one female companion along with the CHW throughout labour and delivery. Several studies have highlighted women experiencing abandonment and neglect during childbirth [29, 61, 62]. Our study also found that some women were left unattended in the delivery room after childbirth for more than 15 minutes. Although few in number, there were instances of women experiencing both verbal and physical abuse at the hands of the provider, mostly during labour and delivery. Similar experiences of disrespectful maternal care including pushing, slapping, shouting, threatening women for refusal to treatment, and unwarranted fundal pressure have been reported in studies from other developing countries including India [22, 27, 28, 61].

Informal payments was also a concern in these facilities. JSY guarantees women delivering at any government institution free delivery care along with monetary incentives. However, research has highlighted issues of informal payments and other out-of-pocket expenditure negating the benefit of the monetary incentive provided by the state [14, 30, 63–65]. While we observed that informal payments were demanded in a few cases, it is possible that the extent of informal payment demanded by the staff is underreported since our presence may have altered their behavior. We also found that providers in many facilities also insisted that patients and their families purchase drugs from private pharmacies located close to the facility, though supplies were available in most facility.

Over the past years there has been a growing pool of evidence from across the globe suggesting that until a satisfactory level of quality is attained in service delivery, the desired and expected reductions in maternal mortality cannot be achieved only by improving infrastructure, accessibility and incentivizing institutional delivery [22, 33, 66]. In India, the current quality assurance guidelines have shifted focus from not only ensuring facility infrastructure, but also addressing measures to ensure patient privacy, increasing patient information and engagement, and setting up a system of grievance redress in the form of Patient Welfare Committee [18]. Our findings have the following policy implications in order to make the health system more outcome driven and responsive to patient’s needs, aspects such as promptness, information sharing and respectful behaviour need to be incorporated in the policies [32, 33]. Studies have highlighted that challenges deterring optimal utilization of facilities includes lack of adherence to clinical protocols; compromised patient safety; women-friendly delivery environment; cognitive support; compromised privacy; frequent abuse and demand for informal payments [22,29]. Identifying and addressing structural gaps, training and orientation of staff towards aspects of person-centered care, and adherence to clinical protocols through criterion-based audits needs to be prioritized and implemented [33, 67]. Training and orientation of staff towards aspects of person-centred care including respectful & supportive care; information sharing; effective communication skills and patient privacy can go a long way in sensitizing providers around person-centred care, supported by public display of behavioural norms in the charter of patient rights and entitlements. The findings from a quality improvement (QI) intervention in public health facilities across six states of India, suggest that simple QI methods like redesign of space and task shifting have the potential to improve quality of facility-based routine clinical practices in resource poor settings like India [62] QI cycles may be followed by performance based grading of facilities on attainment of specific quality benchmarks to encourage adoption of good care practices. A concept of ‘positive deviance approach’ may also be introduced for collating and sharing ‘best practices’ with regards to quality of care in facilities performing better while having access to the same resources and facing similar or worse challenges.

### Limitations

One of the key limitations common to observational studies is that staff actions may have been influenced by the presence of the observers. To minimize this bias, the investigators spent a day in the facility familiarizing themselves with the setting and interacting with the staff before beginning to record the observations. We assumed that this would put the staff at ease with the team by the time the observations began; however, we acknowledge this potential bias remains. The second limitation of the observation method is the observer’s bias in data collection and interpretation of results. We worked to mitigate this by using a standardized checklist to record the data across the team and triangulating the findings of multiple observers.

## Conclusions

It is encouraging to witness developing countries like India slowly realizing the need and importance of improving quality of care in public health facilities in order to make care more responsive to women’s needs. Findings from this study reflect gaps in the ‘quality front’ of maternity care provision in public health facilities in India and our results support the argument for strengthening maternity care services provided by the public health system.

Identifying and addressing structural gaps including infrastructure and medical supplies should be prioritized [34], followed by gaps related to ‘process of care’ including clinical care; patient safety; information sharing; emotional support; informal payments; and disrespectful care. Staff training is required on aspects of person-centered care such as therapeutic communication. Clinical supervision of staff; criterion-based audits; near-miss audits; and supportive supervision should be undertaken for ensuring adherence to clinical care protocols and quality norms [34, 67]. There is evidence that simple Quality Improvement methods like redesigning of space and task shifting have the potential to improve quality of facility-based routine clinical practices in resource-poor settings like India [62]. Appreciating positive deviance and recognizing quality of care ‘best practices’ could also encourage facilities to optimize quality within the given resources.

## Supporting information

S1 Table Profile of study facilities by provider and infrastructure provision. Acronyms: CHC-Community Health Centre; PHC-Primary Health Centre; BPHC-Block Primary Health Centre; FRU-First Referral Unit; ANM-Auxiliary Nurse Midwife. Source: ^$^Observation data and departmental Health Management Information System (HMIS) records, Sep 2016.**Description of data:** The table gives information about profile of the study facilities including facility type; number of in-position staff in the maternity unit; available infrastructure facilities and patient load for vaginal and caesarean deliveries for the month of September 2016.
(DOCX)

S2 Table Checklist for facility observation. (DOCX)

## Abbreviations

CHW: Community Health Worker
FHR: Fetal Health Rate
JSY: Janani Suraksha Yojana
MDGs: Millennium Development Goals
MIL: Mother-In-Law
MMR: Maternal Mortality Ratio
MOIC: Medical Officer In-Charge
NHM: Nation Health Mission
NMR: Neo-natal Mortality Rat
PCC: Person Centred Care
PNC: Post-Natal Care
QoC: Quality of Care
SDGs: Sustainable Development Goals
UP: Uttar Pradesh
WHO: World Health Organization
