# Supplementary material for: Is quality of care during childbirth consistent from admission to discharge? A qualitative study of delivery care in Uttar Pradesh, India
Source: PLoS One. 2018 Sep 27;13(9):e0204607. doi: 10.1371/journal.pone.0204607 (PMC6160099; doi:10.1371/journal.pone.0204607)
Supplement: S1 Table — Acronyms: CHC-Community Health Centre; PHC-Primary Health Centre; BPHC-Block Primary Health Centre; FRU-First Referral Unit; ANM-Auxiliary Nurse Midwife. Source: $Observation data and departmental Health Management Information System (HMIS) records, Sep 2016.Description of data: The table gives information about profile of the study facilities including facility type; number of in-position staff in the maternity unit; available infrastructure facilities and patient load for vaginal and caesarean deliveries for the month of September 2016. (DOCX) [file pone.0204607.s001.docx]

**S1 Table. Profile of study facilities by provider and infrastructure provision.** Acronyms: CHC-Community Health Centre; PHC-Primary Health Centre; BPHC-Block Primary Health Centre; FRU-First Referral Unit; ANM-Auxiliary Nurse Midwife

Source: ^$^Observation data and departmental Health Management Information System (HMIS) records, Sep 2016.

**Description of data:** The table gives information about profile of the study facilities including facility type; number of in-position staff in the maternity unit; available infrastructure facilities and patient load for vaginal and caesarean deliveries for the month of September 2016.

| **Districts** | **District 1** | | | | | | | **District 2** | | | |
| --- | --- | --- | --- | --- | --- | --- | --- | --- | --- | --- | --- |
| **Facility** | Facility 1 | Facility 2 | | Facility 3 | | Facility 4 | Facility 5 | Facility 6 | Facility 7 | Facility 8 | Facility 9 |
| **Facility type** | Secondary- CHC | Primary- PHC | | Secondary- CHC/FRU | | Primary- BPHC/PHC | Primary-BPHC/PHC | Secondary- CHC | Secondary- CHC | Secondary- CHC | Secondary- CHC |
| **HR in position** | | | | | | | | | | | |
| Lady Medical Officer | 1 | 2 | | 3 | | 2 | 1 | 4 | 3 | 5 | 2 |
| Nurses and ANM | 7 | 4 | | 4 | | 5 | 4 | 9 | 5 | 8 | 9 |
| Other support Staff | 2 | 3 | | 2 | | 2 | 2 | 1 | 2 | 3 | 2 |
| **Infrastructure Provision#** | | | | | | | | | | | |
| No. of beds | 7 | 5 | | 24 | | 5 | 7 | 4 | 7 | 12 | 4 |
| No. of Delivery Table | 3 | 3 | | 2 | | 2 | 3 | 3 | 2 | 2 | 3 |
| Pharmacy and Laboratory | Yes | Yes | | Yes | | Yes | Yes | Yes | Yes | Yes | Yes |
| Hospital Meals | No | Yes | | No | | Yes | No | No | Yes | No | Yes |
| Newborn Stabilization Unit/corner | Not functional | Functional | | Functional | | Not functional | Not functional | Functional | Functional | Functional | Not functional |
| Electricity with backup | Yes | Yes | | Yes | | Yes | Yes | Yes | Yes | Yes | Yes |
| Drinking Water | No | No | | No | | No | Yes | No | No | Yes | No |
| **No. of Deliveries conducted, September 2016 ^$^** | | | | | | | | | | | |
| Vaginal | 228 | | 110 | | 219 | 166 | 142 | 210 | 154 | 257 | 127 |
| C-section | 0 | | 0 | | 0 | 0 | 0 | 5 | 0 | 4 | 4 |
