# Supplementary material for: Is quality of care during childbirth consistent from admission to discharge? A qualitative study of delivery care in Uttar Pradesh, India
Source: PLoS One. 2018 Sep 27;13(9):e0204607. doi: 10.1371/journal.pone.0204607 (PMC6160099; doi:10.1371/journal.pone.0204607)
Supplement: S2 Table — (DOCX) [file pone.0204607.s002.docx]

**S2 Table : Checklist for facility Observation**

| **Facility** |  | **District** |  |
| --- | --- | --- | --- |
| **Observation No.** |  | **Date** |  |
| **Observer Name** |  | **Start Time** |  |
| **Provider Name (with designation)** |  | **End Time** |  |
| **Form Verified By** |  | **Gravida** |  |

| **Section A: Admission Process** | | | |
| --- | --- | --- | --- |
| ***Sl. No.*** | ***Items*** | ***Response***  ***(Yes/No)*** | ***Notes*** |
| A.1 | Any signboard showing the registration counter? |  |  |
| A.2 | Ramp/clear way to registration counter |  |  |
| A.3 | Availability of personnel at registration counter |  |  |
| A.4 | How long did it take the woman to get registered (specify time)? |  |  |
| A.5 | Did anyone accompany the woman? |  |  |
| A.6 | Who accompanied the woman (family member/ASHA/other)? Please record in Notes column. |  |  |
| A.7 | Did the woman have a place to sit? |  |  |
| A.8 | Did the woman receive any card/token/receipt? |  |  |
| A.9 | Was any information provided to women/ family members during registration? If yes, note the type of information shared. |  |  |
| A.10 | Did anyone (provider/sweeper/other facility staff) ask the woman or those who accompanied her for any money (informal payment/bribe/gift etc.) before her admission process was completed? (Specify amount in Notes column) |  |  |
| A.11 | Observe:  Was there a queue……………………………………………….  Defined waiting area…………………………………………….  Any seating arrangement……………………………………..  Drinking water provision……………………………………..  Cleanliness…………………………………………………………….  Availability of power supply (fan+ light)………………… |  |  |

**Observer Remarks**

………………………………………………………………………………………………………………………………………………………………………………………………………………………………………………………………………………………………………………………………………………………………………………………………………………………………………………………………………………………………………………

| **Section B: Pre-Procedure** | | | |
| --- | --- | --- | --- |
| ***Sl. No.*** | ***Items*** | ***Response***  ***(Yes/No)*** | ***Notes*** |
| B.1 | How long did the woman have to wait for the initial examination to take place? (record in minutes) |  |  |
| B.2 | Who completed the woman’s initial examination?  (Doctor/Nurse/Other) Please note designation and gender. |  |  |
| B.3 | Did the provider evaluate the general condition of the woman **(within the first hour and then once every 4 hours depending on how long she was in labour)** | | |
|  | Blood pressure  Pulse  Temperature  Test for glucose in urine  Test for hemoglobin  Any other test ………………………….. |  |  |
|  |  |  |  |
|  |  |  |  |
|  |  |  |  |
|  |  |  |  |
|  |  |  |  |
| B.4 | Diagnosis and monitoring of labour during initial exam: | | |
|  | Check antenatal card |  |  |
|  | Asked about onset of labour |  |  |
|  | Asked if membrane had ruptured/her water had broken |  |  |
|  | Ask if she had vaginal discharge |  |  |
|  | Ask how she was feeling |  |  |
|  | Determine position of fetus |  |  |
|  | Ask if she was bleeding |  |  |
|  | Measure fetal heart rate |  |  |
|  | Vaginal examination |  |  |
|  | Time contractions |  |  |
|  | Ask if she had any questions |  |  |
| B.5 | Did the care provider wash hands before examination? |  |  |
| B.6 | Did the care provider clean woman’s perineum before examination? |  |  |
| B.7 | Was the woman’s privacy maintained during the physical examination (was there a curtain, separate room etc.)? |  |  |
| B.8 | Did someone accompany her during the initial examination? |  |  |
| B.9 | Was the woman told how far along she was in labour? (centimeters dilated) |  |  |
| B.10 | Were the family members informed about the time stage of labour/ or if there was any complication? |  |  |
| B.11 | Was the woman or her family able to ask any question of the providers? |  |  |
| B.12 | If yes, what/how was the response of the providers?  ……………………………………………………………………………………………………………………………………………………………………………………………………………………………………………………………………………………………………………………………………………………………………………………………………………………………… | | |
| B.13 | Was she was shifted to the observation ward? |  |  |
| B.14 | If yes to B.13, Did she get her own bed? |  |  |
| B.15 | If yes to B.13, How long did she have to wait at the observation ward? |  |  |
| B.16 | If yes to B.13, Was she was alone in the observation ward? |  |  |
| B.17 | How was the women was shifted from observation ward to labour room (she walked/ trolley/ other)? |  |  |
| B.18 | Any health provider examined/enquired about her condition at at least 4 hour intervals? |  |  |
| B.19 | If yes to B.17, Who and what all examined/enquired?  ……………………………………………………………………………………………………………………………………………………………………………………………………………………………………………………………………………………………………………………………………………………………………………………………………………………………………………………………………………………………………………………………………………………………………………………………………………………………………………………………………………………………………………………………….................................................................................................................................................................................... | | |
| B.20 | Did anyone (provider/sweeper/other facility staff) ask the woman or those who accompanied her for any money (informal payment/bribe/gift etc.) before her initial examination was completed? (Specify amount in Notes column) |  |  |

**Observer’s Comments:**

…………………………………………………………………………………………………………………………………………………………………………………………………………………………………………………………………………………………………………………………………………………………………………………………………………………………………………………………………………………………………………………………………………………………………………………………………………………………………………………………………………………………………………………………………………………………….……………………………………………………………………………………………………………………………………………………………………………………………………………………………………………………………………………………………………………………………………………………………………………………

| **Section C: Procedure** |
| --- |

| ***Sl. No.*** | ***Items*** | ***Response***  ***(Yes/No)*** | ***Notes*** |
| --- | --- | --- | --- |

| C.1 | How much time did the woman have to wait before being shifted to the delivery room? (Please note the time, who decided)  …………………………………………………………………………………………………………………………………………………………………………………………………………………………………………………………………………………………………………………………….  ……………………………………………………………………………………………………………………………………………………………………………………………………………………………………………………………………………………………………………………………. | | |
| --- | --- | --- | --- |
| C.2 | Did the care provider help patient to climb on the delivery table? |  |  |
| C.3 | Did the care provider explain to the woman how to lie down on delivery table?  Note if the woman appeared uncomfortable in the position in which she was delivering. |  |  |
| C.4 | Was the woman left alone at any point?  If Yes, record how long in the notes column. |  |  |
| C.5 | Who did the delivery? (Doctor/Nurse/Other) Note the designation, gender of the provider and if they were present through the entire process. |  |  |
| C.6 | Was there any one who assisted?  If yes, note the designation and their role. |  |  |
| C.7 | Did the provider wash his/her hands with soap and water or used alcohol hand rub before any examination of woman? |  |  |
| C.8 | Did the provider wears sterile surgical gloves? |  |  |
| C.9 | Did they use box of sterile instruments for each delivery? |  |  |
| C.10 | Whether the instruments are soaked in decontamination solution? |  |  |
| C.11 | Monitoring of labour – record keeping………………..  Partograph filled during labour …………………………..  Partograph filled after delivery…………………………… |  |  |
| C.12 | Performs episiotomy if indicated |  |  |
| C.13 | Motivated woman to push the baby |  |  |
| C.14 | Record time of the delivery of the baby |  |  |
| C.15 | Administers uterotonic?  (Name, time, route) …………………………………………………………………………………………………………………………………………………………………………………………….......................................................................................................................................................................................................................................................................................................... | | |
| C.16 | Did they disinfect the skin before injection? |  |  |
| C.17 | Performs uterine massage immediately following the delivery of the placenta |  |  |
| C.18 | Assesses completeness of the placenta and membranes |  |  |
| C.19 | Assesses for perineal and vaginal lacerations |  |  |
| C.20 | Was someone present with the woman to provide support? Please note who this person was (family/ASHA/other) |  |  |
| C.21 | Was the woman unnecessarily exposed at any point during the delivery? |  |  |
| C.22 | Was there a dividing screen/curtain between delivery tables?  (Delivery was not seen by other patients) |  |  |
| C.23 | Did the woman/companion have to arrange for any medicine/cotton etc? (Elaborate) ………………………………………………………………………………………………………………………………………………………………………………………………………………………………………………………………………………………………………………………………………………………………………………………………………………………………………………………………………………………………………………………………………………………………………………………………………………………………………………………………  ………………………………………………………………………………………………………………………………………............................................................................................................................................................................................. | | |
| C.24 | Did the woman face any abuse during labour? (physical or verbal/specify the provider/staff)  ……………………………………………………………………………………………………………………………………………………………………………………………………………………………………………………………………………………………………………………………………………………………………………………………………………………………………………………………………………………………………………………………………………………………………………………………………………………………………………………………… | | |
| C.25 | Observe –   - The cleanliness of the delivery room - Availability of separate delivery tables - Cleanliness of delivery tables - Availability of power supply (fan+ light) |  |  |
| C.26 | Did anyone (provider/sweeper/other facility staff) ask the woman or those who accompanied her for any money (informal payment/bribe/gift etc.) before or during her delivery? (Specify amount in Notes column) |  |  |
| C.27 | Was a blood transfusion required? |  |  |
| C.28 | If yes, who arranged and from where? |  |  |
|  | **Immediate postpartum care** | | |
| C.29 | Takes mother's vital signs 15 minutes after birth |  |  |
| C.30 | Palpates uterus 15 minutes after delivery of placenta |  |  |
| C.31 | Administers antibiotics to mother postpartum if indicated (state reason and name of antibiotics)  ………………………………………………………………………………………………………………………………………………………………………………………………………………………………………………………………………………………………………………………………………… |  |  |
| C.32 | Was the woman left alone in the delivery room after her delivery? (If yes, please note duration) |  |  |
|  | **Waste disposal** |  |  |
| C.33 | Disposal of all sharps in a puncture-proof container immediately after use |  |  |
| C.34 | Disposal of all contaminated waste in leak-proof containers |  |  |
|  | **Physical Examination of the Baby** | | |
| C.35 | Clear nose and mouth |  |  |
| C.36 | Apply antimicrobial ointment in eye |  |  |
| C.37 | Disinfect cord |  |  |
| C.38 | Cover baby |  |  |
| C.39 | Immediate skin-to skin contact |  |  |
| C.40 | Was the baby was shown to the mother after delivery? |  |  |
| C.41 | When was the breastfeeding started? …………………………………………………………………………. |  |  |
| C.42 | Checks baby's temperature 15 minutes after birth |  |  |
| C.43 | Checks baby's skin color 15 minutes after birth |  |  |
| C.44 | Administers Vitamin K to newborn |  |  |
| C.45 | If newborn resuscitation needed, then elaborate the procedure followed. [or any other complication] ……………………………………………………………………………………………………………………………………………………………………………………………………………………………………………………………………………………………………………………………………………………………………………………………………………………………………………………………………………………………………………………………………………………………………………………………………………………………………………………………………………………………………………………………………………………………………………………………………………………………… | | |

**Observer’s Comments:**

…………………………………………………………………………………………………………………………………………………………………………………………………………………………………………………………………………………………………………………………………………………………………………………………………………………………………………………………………………………………………………………………………………………………………………………………………………………………………………………………………………………………………………………………………………………………….…………………………………………………………………………………………………………………………………………………………………………………………………………………………………………

| **Section D: Post-Procedure** |
| --- |

| ***Sl. No.*** | ***Items*** | ***Response***  ***(Yes/No)*** | ***Notes*** |
| --- | --- | --- | --- |

| D.1 | When was the woman shifted to the PNC ward? (wait-time after birth) |  |  |
| --- | --- | --- | --- |
| D.2 | How was she transported (wheel chair/stretches/any other)  …………………………………………………………………………………………………………………………………………………………………………………………………………………………………………………………………………………………………… | | |
| D.3 | Observe –   - The cleanliness of PNC ward - Availability of cot/bed/pillow - Cleanliness of bed/bed sheet - Clean toilets - Clean bathrooms - Availability of water in toilet and bathrooms - Availability of drinking water - Curtains in PNC ward - Presence of security to guard the ward/regulate visitors - Male visitors in the ward - Availability of power supply (fan+ light) |  |  |
| D.4 | Whether the woman and baby got a separate cot/bed? |  |  |
| D.5 | Did the companion have a separate bed/cot/other seating? |  |  |
| D.6 | Did the companion stay throughout in the PNC ward? |  |  |
| D.7 | How often did the Doctor/Provider visit the ward? |  |  |
| D.8 | During visits, did the doctor examine the woman? |  |  |
| D.9 | What examinations were conducted during the providers visit to the PNC ward?   1. Check blood pressure at least once 2. ……………………………………………………………… 3. ……………………………………………………………. 4. ……………………………………………………………... 5. ……………………………………………………………… |  |  |
| D.10 | Any pediatrician examined the baby at the facility/baby was taken to any pediatrician? |  |  |
| D.11 | What examinations were conducted for the baby?   1. …………………………………………………………….. 2. …………………………………………………………….. 3. ……………………………………………………………… 4. ……………………………………………………………… 5. ……………………………………………………………… |  |  |
| D.12 | If C-section, when were the stitches removed? |  |  |
| D.13 | Was there a health provider available 24*7 in the PNC ward?Elaborate………………………………………………………………………………………………………………………………………………………… |  |  |
| D.14 | Did the provider share information about mother and baby’s health to the family members and to the women? |  |  |
| D.15 | If yes, did it appear as though the women/ family member understood this information? |  |  |
| D.16 | Was the provider present in the ward respond to woman’s requests? |  |  |
| D.17 | Did the provider respond politely to woman’s queries? |  |  |
| D.18 | Whether the facility provided food to the woman?  Explain……………………………………………………………………..…………………………………………………………………………………….. |  |  |
| D.19 | Did the women face any abuse (verbal/ physical) from the provider / cleaner etc.? Please elaborate. |  |  |
| D.20 | Did the women/companion have to buy any medicine/supplies during her stay at the PNC? |  |  |
| D.21 | Did the woman receive counseling regarding:   1. Family planning   ………………………………………………………………   1. Immunization of child ……………………………………………………………… 2. Exclusive breast feeding ……………………………………………………………… 3. Danger signs of baby’s health ……………………………………………………………... 4. Danger signs for mother’s health ………………………………………………………………   Any other ………………………………………………………… |  |  |
| D.22 | Did the woman develop any complications after the delivery? |  |  |
| D.23 | If yes, what was the diagnosis?  Elaborate the procedures undertaken at the facility  ………………………………………………………………………………………………………………………………………………………………………………………………………………………………………………………………………………………………………………………………………………………………………………………………………………………………………………………………………………………………………………………………………………………………………………………………………… | | |
| D.24 | Did the baby develop any condition that required medical attention? |  |  |
| D.25 | If yes, elaborate ………………………………………………………………………………………………………………………  ………………………………………………………………………………………………………………………………………………..  ………………………………………………………………………………………………………………………………………………………………………………………………………………………………………………………………………………………………….. | | |
| D.26 | Did anyone (provider/sweeper/other facility staff) ask the woman or those who accompanied her for any money (informal payment/bribe/gift etc.) after her delivery, while she was in the PNC ward? (Specify amount in Notes column) |  |  |

**Observer’s Comments:**

……………………………………………………………………………………………………………………………………………………………………………………………………………………………………………………………………………………

| **Section E: Discharge** |
| --- |

| ***Sl. No.*** | ***Items*** | ***Response***  ***(Yes/No)*** | ***Notes*** |
| --- | --- | --- | --- |

| E.1 | Did the woman receive counseling regarding:   1. Family planning   ………………………………………………………………   1. Immunization of child ……………………………………………………………… 2. Exclusive breast feeding ……………………………………………………………… 3. Danger signs of baby’s health ……………………………………………………………... 4. Danger signs for mother’s health ……………………………………………………………… 5. Any other ………………………………………………………………. |  |  |
| --- | --- | --- | --- |
| E.2 | Did the woman’s family receive counseling regarding:   1. Family planning   ………………………………………………………………   1. Immunization of child ……………………………………………………………… 2. Exclusive breast feeding ……………………………………………………………… 3. Danger signs of baby’s health ……………………………………………………………... 4. Danger signs for mother’s health ……………………………………………………………… 5. Any other ……………………………………………………………… |  |  |
| E.3 | Who provided the information / counseling? ………………………………………………………………………………………………………………………………………………… | | |
| E.4 | When and where (place) was the information/counseling provided? ………………………………………………………………………………………………………………………………………………… | | |
| E.5 | Was the counseling location crowded? |  |  |
| E.6 | Was the woman/or those who accompanied her given instructions for follow-up check-ups? |  |  |
| E.7 | Was immunization schedule and related information given? |  |  |
| E.8 | Did the woman receive JSY cash/done paper work during the time of discharge? |  |  |
| E.9 | Did she have to pay for the services (laundry/sweeper etc.)? |  |  |
| E.10 | Did she receive any medicine/cotton etc. from the facility when being discharged? |  |  |
| E.11 | Was the woman’s transport home arranged by the facility (ambulance, or other)? |  |  |
| E.12 | Did anyone (provider/sweeper/other facility staff) ask the woman or those who accompanied her for any money (informal payment/bribe/gift etc.) before/at discharge? (Specify amount in Notes column) |  |  |

**Observer’s Comments:**

…………………………………………………………………………………………………………………………………………………………………………………………………………………………………………………………………………………………………………………………………………………………………………………………………………………………………………………………………………………………………………………………………………………………………………………………………………………………………………………………………………………………………………………………………………………………….……………………………………………………………………………………………………………………………………………………………………………………………………………………………………………………………………………………………………………………………………………………………………………………………………………………………………………………………………………………………………………………………………………………………………………………………………………………………………………………………
